# Monitoring Risk: Tick and *Borrelia burgdorferi* Public Participatory Surveillance in the Canadian Maritimes, 2012–2020

**DOI:** 10.3390/pathogens10101284

**Published:** 2021-10-06

**Authors:** Julie Lewis, Andrea M. Kirby, Kami Dawn Harris, Cory L. Filiaggi, Alexandra Foley-Eby, Malcolm Mann, David Lieske, Vett K. Lloyd

**Affiliations:** 1 Department of Biology, Mount Allison University, Sackville, NB E4L 1G7, Canada; jlewis@mta.ca (J.L.); amkirby@mta.ca (A.M.K.); sgreenwood@upei.ca (K.D.H.); corey.filiaggi@iwk.nshealth.ca (C.L.F.); fxu874@usask.ca (A.F.-E.); mgmann@mta.ca (M.M.); 2 Département de Chimie et Biochimie, Université de Moncton, Moncton, NB E1A 3E9, Canada; 3 Department of Pathology and Microbiology, Atlantic Veterinary College, University of Prince Edward Island, Charlottetown, PE C1A 4P3, Canada; 4 IWK Health Centre, Halifax, NS B3K 6R8, Canada; 5 Department of Veterinary Microbiology, University of Saskatchewan, Saskatoon, SK S7N 5B4, Canada; 6 Department of Geography and Environment, Mount Allison University, Sackville, NB E4L 1G7, Canada; dlieske@mta.ca

**Keywords:** ticks, surveillance, *Ixodes scapularis*, *Ixodes cookei*, *Dermacentor variabilis*, Canada, *Borrelia*

## Abstract

Ticks are vectors of many diseases, including Lyme disease (Ld). Lyme disease is an emerging disease in Canada caused by infection with the Lyme borreliosis (Lb) members of the *Borrelia* genus of spirochaete bacteria, of which *Borrelia* *burgdorferi* is regionally the most prevalent. The primary tick vector in central and eastern Canada, *Ixodes scapularis*, is increasing in numbers and in the geographical extent of established populations. This study documents the distribution of ticks recovered by passive surveillance, and their *B.* *burgdorferi* infection prevalence, in three Canadian Maritime provinces from 2012–2020. These regions represent areas in which tick populations are widely established, establishing, and considered non-established. Using a community science approach by partnering with veterinarians and members of the public, we collected over 7000 ticks from the 3 provinces. The three species found most often on companion animals and humans were *I. scapularis* (76.9%), *Ixodes cookei* (10.4%) and *Dermacentor variabilis* (8.9%). The most common hosts were dogs (60.5%), cats (16.8%) and humans (17.6%). As is typical of passive surveillance tick collections, the majority of ticks recovered were adult females; for *I. scapularis* 90.2%, 5.3%, 3.9% and 0.6% of the total of 5630 ticks recovered for this species were adult females, adult males, nymphs and larvae, respectively. The majority of *B. burgdorferi*-infected ticks were *I. scapularis*, as expected. *Borrelia* infection prevalence in *I scapularis* was higher in Nova Scotia (20.9%), the province with the most endemic regions, than New Brunswick (14.1%) and Prince Edward Island (9.1%), provinces thought to have established and non-established tick populations, respectively. The province-wide *Borrelia* infection prevalence generally increased in these latter tow provinces over the course of the study. The host did not have a significant effect on *B. burgdorferi* infection prevalence; *I. scapularis* ticks from dogs, cats, humans was, 13.3% (n = 3622), 15.6% (n = 817), 17.9% (n = 730), respectively. No *I. scapularis* larvae were found infected (n = 33) but *B. burgdorferi* was detected in 14.8% of both adults (n = 5140) and nymphs (n = 215). The incidence of *B. burgdorferi* infection also did not differ by engorgement status 15.0% (n = 367), 15.1% (n = 3101) and 14.4% (n = 1958) of non-engorged, engorged and highly engorged ticks, respectively, were infected. In New Brunswick, at the advancing front of tick population establishment, the province-wide infection percentages generally increased over the nine-year study period and all health district regions showed increased tick recoveries and a trend of increased percentages of *Borrelia*-infected ticks over the course of the study. Within New Brunswick, tick recoveries but not *Borrelia* infection prevalence were significantly different from endemic and non-endemic regions, suggesting cryptic endemic regions existed prior to their designation as a risk area. Over the 9 years of the study, tick recoveries increased in New Brunswick, the primary study region, and *I. scapularis* recoveries spread northwards and along the coast, most but not all new sites of recoveries were predicted by climate-based models, indicating that ongoing tick surveillance is necessary to accurately detect all areas of risk. Comparison of tick recoveries and public health risk areas indicates a lag in identification of risk areas. Accurate and timely information on tick distribution and the incidence of *Borrelia* and other infections are essential for keeping the public informed of risk and to support disease prevention behaviors.

## 1. Introduction

Ticks are obligate blood-feeding ectoparasites that are able to transmit a greater array of pathogens than any other arthropod [1]. As ticks feed, nutrients from the host’s blood are absorbed, excess water is removed by the tick salivary glands and secreted back into the host [2]. During this process, a variety of microorganisms including bacteria, viruses, protozoa, fungi, and nematodes, some of which are pathogenic, can be transferred to the host [3,4]. In this way, ticks can vector many disparate pathogens, producing a variety of diseases. In temperate regions of the world, the most common of these is Ld, or Lb. Ld is caused by some species of the Lb group, or *Borrelia burgdorferi* sensu lato complex [5], although only *Borrelia burgdorferi* sensu stricto is routinely monitored in North America [6,7]. Ld is marked by a panoply of multisystemic symptoms that affect the musculoskeletal, respiratory, circulatory, nervous, and other systems, which can have debilitating or in some cases fatal, health consequences [7,8,9,10,11].

In eastern Canada, the tick species that most often parasitize humans and companion animals include *Ixodes scapularis* (Say, 1821) also known as the blacklegged or deer tick, *Ixodes cookei* (Packard, 1869) or the groundhog/woodchuck tick, and *Dermacentor variabilis* (Say, 1821) or the American dog tick [12]. All of these ticks can vector a variety of pathogens including *Borrelia* spirocheates [12], which are efficiently vectored by *I. scapularis* [13] and, possibly less efficiently, by *I. cookei* [14,15]. Both species also transmit other pathogens including the Powassan virus/Deer Tick Virus, which causes an infection of the central nervous system resulting in encephalitis and meningitis [16]. *D. variabilis*, the American dog tick or wood tick, is not thought to be a competent vector of *B. burgdorferi* but can transmit *Francisella tularensis*, the bacteria responsible for tularemia, *Rickettsia rickettsii*, the bacteria responsible for Rocky Mountain spotted fever, *Ehrlichia* *chaffeensis*, the bacteria responsible for Ehrlichiosis, *Anaplasma marginale*, the bacteria responsible for Anaplasmosis or tick-born fever in a variety of mammals, and likely related pathogens [17,18,19,20].

The tick species that parasitize humans and companion animals do so because they are generalist feeders, so are not overly meticulous in choosing their hosts [12]. As a consequence, many mammalian host species are available to support the expanding populations of *I. scapularis, D. variabilis* and other introduced tick species [21]. Further, many of these species are effective reservoir hosts for *B. burgdorferi,* thus perpetuating the infection cycle. Reservoir species in the Canadian Maritimes include a wide variety of rodents such as deer mice and white-footed mice, shrews, squirrels, chipmunks, and wood rats [6,22]. Some bird species are also effective reservoirs [23], in addition to their important role in tick dispersal. Each spring, an estimated 50 to 175 million *I. scapularis* are dispersed into Canada by migratory birds [24], with some species carrying *I. scapularis* long distances northwards [25,26,27]. Once introduced, both biotic and abiotic factors influence the proportion of ticks able to feed and survive long enough to reproduce and establish new populations [28]. Surveillance efforts have revealed a northward expansion of established *I. scapularis* and *D. variabilis* populations in response to complex and inter-related changes in climate, mammal and bird migrations, rodent populations, landscape fragmentation and the resultant effect on biodiversity, the nature of vegetation and forests and foliage type, wildlife management practices, human demographics, and other anthropomorphic factors [21,29,30]. 

Humans and companion animals have lifestyles that make the repeated contact with ticks, needed to maintain tick or *Borrelia* populations, unlikely, and are, therefore, considered accidental hosts rather than reservoirs. Thus, barring congenital transmission of *Borrelia* [31], humans are not ecologically relevant in perpetuating *Borrelia* in the environment. Nevertheless, *Borrelia* infections can result in Ld and are of considerable importance in both human and veterinary medicine. Tick surveillance plays an important role in disease diagnosis and subsequent treatment; clinical and serological evidence is considered in light of a history of exposure to known tick endemic areas [32]. Without known exposure, positive serology results risk being discounted by invocation of pre-test probability considerations [33]. 

Tick surveillance allows monitoring of *I. scapularis* distribution and *B. burgdorferi* infection prevalence. How the surveillance is performed affects its sensitivity. Active surveillance involves capturing ticks from the environment, whereas passive surveillance involves collecting ticks from humans and companion, agricultural, or wild animals [34]. Both collection methods have strengths and weaknesses [34]. Active surveillance suffers from low sensitivity. It is less efficient for a single team to find ticks in the field during a short site visit than having veterinarians and members of the public involved in the collection process over a period of years. Additionally, ticks are only captured from the environment when actively questing for a blood meal and appropriate conditions for this activity may not occur at the time of site visit. Finally, field collection of ticks relies on ticks being captured by an inert “tick drag” that does not mimic a host in a biologically meaningful manner, although adding a source of CO_2_ or host body odor to the sheet has been shown to improve tick collection for a European tick species [35]. All these factors contribute to reduced sensitivity of this surveillance method. This can lead to an underestimation of tick populations, particularly in areas with low tick density as occurs in areas with emerging tick populations, making this an unsuitable surveillance method in such situations. However, active surveillance does permit precise geographical location of recovered ticks, recording of ecosystem variables and has the advantage of allowing direct comparison of tick density between populations as search effort is not influenced by proximity to human communities so it is valuable for ecological studies in endemic areas [36]. Passive collection, often involving a community science approach, is able to collect more ticks over a larger geographic area with fewer logistical challenges than active surveillance [37]. Passive surveillance has been criticized for being too sensitive as it can recover both endemic ticks from local populations and adventitious ticks dropped outside of endemic areas by migratory animals [34]. Nonetheless, as the health risk from ticks stems from both home-grown ticks in endemic locations and introduced adventitious ticks [36], this is, in fact, a strength of passive surveillance in the context of risk assessment. Passive surveillance also provides the capacity for sensitive monitoring of the continually evolving endemic areas. Ultimately, the impact of these relative advantages and disadvantages depends on the research question being addressed, however in a public health setting, the lower cost and increased sensitivity associated with passive surveillance are considerable advantages. 

Comprehensive surveillance in areas outside of known endemic regions is critical for monitoring the continually evolving locations and size of tick populations [38]. The three Canadian Maritime provinces of Nova Scotia (NS), New Brunswick (NB) and Prince Edward Island (PEI), while geographically connected, differ in respect to the establishment of populations of the introduced *I. scapularis*, *I. cookei* and *D. variabilis* tick species. Nova Scotia is the southernmost province and, during the study period of 2012–2020, known endemic and Ld risk areas expanded from the southwest of the province across the entire province [39]. During the same time period, the known endemic and risk areas in New Brunswick expanded from two localized populations in the southwest to the southern third of the province [40,41]. Prince Edward Island has been considered to have no endemic tick populations, although ticks that are presumably adventitious have been reported on the island since 1989 [42]. In this study, we report passive surveillance tick recoveries and *B. burgdorferi* infection prevalence from these three neighboring provinces during a 9-year period from 2012–2020. Our results show an expanding risk for Ld outside of known and suspected endemic sites, particularly in riparian and coastal regions. 

## 2. Results

### 2.1. Tick Submissions 

The number of ticks submitted over the course of the study increased over time; 365, 588, 774, 792, 1026, 1350, 814, 1181, and 433 were submitted from 2012–2020, respectively, for a total of 7323 (Table 1). The reduced number of submissions in 2020 corresponds with transition to cost-recovery versus free tick testing. The most commonly submitted tick species, all years combined, were *I. scapularis* (76.9%; 5630/7323)*, I. cookei* (10.4%; 761/7323) and *D. variabilis* (8.9%; 655/7323) (Table 1). Other less frequently recovered species included *Haemaphysalis leporispalustris* (Packard, 1869) (rabbit tick), *Ixodes muris* (Bishopp and Smith, 1937) (mouse tick), *Dermacentor albipictus* (Packard, 1869) (moose tick), *Ixodes marxi* (Banks, 1908) (squirrel tick), *Ixodes uriae* (White, 1852) (seabird tick), *Rhipicephalus sanguineus* (Latreille, 1806) (brown dog tick) which collectively comprised 0.9% of recoveries (66/7323). As expected for passive surveillance, and consistent with similar studies [43,44,45,46]; most submissions were of adult females, for all tick species (Table 1). For *I. scapularis* 90.2%, 5.3%, 3.9% and 0.6% of the total of 5630 ticks recovered for this species were adult females, adult males, nymphs and larvae, respectively. For *I. cookei*, 54.4%, 1.3%, 35% and 9.3% of the 761 recovered ticks of this species were adult females, adult males, nymphs and larvae, respectively. For *D. variabilis*, 62.3%, 33.6%, 2.6% and 1.5% of the 655 recovered ticks of this species were adult females, adult males, nymphs and larvae, respectively. The most common hosts contributing ticks to this study were dogs (60.5%), cats (16.8%) and humans (17.6%) (Table 1). Additionally, ticks were also recovered unfed from the field, after detaching from an unknown host when fully engorged, from unspecified hosts and also from wildlife and agricultural hosts (cows, pigs, horses, groundhogs, coyotes, foxes, moose, bear, deer, rabbits, hares, seabirds, songbirds, mice, voles, skunks, squirrels, raccoons and shrews) (Table 1). This study was promoted exclusively in New Brunswick and 78.9% of ticks came from the province. However, ticks were also received from the other Canadian provinces including Nova Scotia (17.2%), Ontario (1.1%), Prince Edward Island (1.0%), Quebec (0.2%), Alberta (0.2%), British Columbia (<0.1%), Manitoba (<0.1%), Saskatchewan (<0.1%) (Table 2). Additionally, ticks were received from other countries in North America, South America, Europe, Africa, Asia and Australia (0.9%) and from unspecified locations (0.7%). Submissions from Prince Edward Island were low in the earlier years of this study, reflecting a local tick surveillance program led by the Island veterinary community [44]. When the program was discontinued, tick submissions to this study increased and a targeted passive surveillance program on Prince Edward Island in 2016 and 2017 retrieved 445 ticks, 97.8% of which were *I. scapularis* [47]. This suggests that the low recoveries from Prince Edward Island reflects lack of local targeted surveillance rather than lack of ticks.

**Figure 1:**
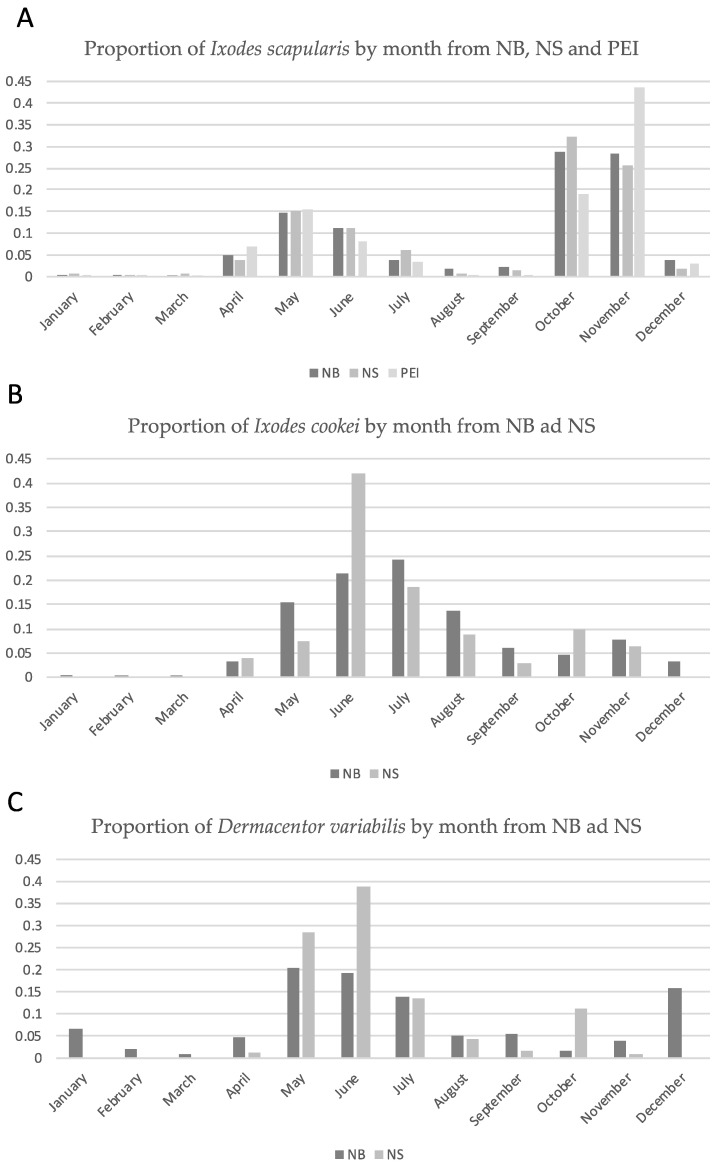
A comparison of seasonal recoveries of *Ixodes scapularis* (**A**), *Ixodes cookei* (**B**) and *Dermacentor variabilis* (**C**) between New Brunswick (NB), Nova Scotia (NS) and Prince Edward Island (PEI). The proportion of the annual tick submissions, per province, is show by month of collection. There were insufficient *Ixodes cookei* and *Dermacentor variabilis* recoveries from Prince Edward Island (PEI) for comparison.

The seasonal distribution of *I. scapularis, I. cookei* and *D. variabilis* submissions is shown in Figure 1. The seasonal recovery pattern is similar between collection years (Supplemental Figure S1) and between Maritime provinces (Figure 1). *I. scapularis* was consistently recovered in all months except February, but most were submitted within two peak periods, the months of April to July and October to November; the spring and fall tick seasons, respectively (Figure 1). The seasonal recovery of ticks from Prince Edward Island has been found to be similar [47]. The seasonal distribution of *I. cookei* and *D. variabilis* submissions from New Brunswick and Nova Scotia were also similar between collection years (Supplemental Figure S2); there were insufficient submissions from Prince Edward Island for meaningful comparison. *I. cookei* was consistently recovered in all months except February and March but, unlike *I. scapularis*, collection during the fall was modest with most submissions occurring from May to August. *D. variabilis* donations were received in all months of the year with a peak from May to July but an appreciable number of specimens were collected during the winter months as well. 

### 2.2. Geographic Distribution of I. scapularis Ticks and Borrelia burgdorferi

**Figure 2:**
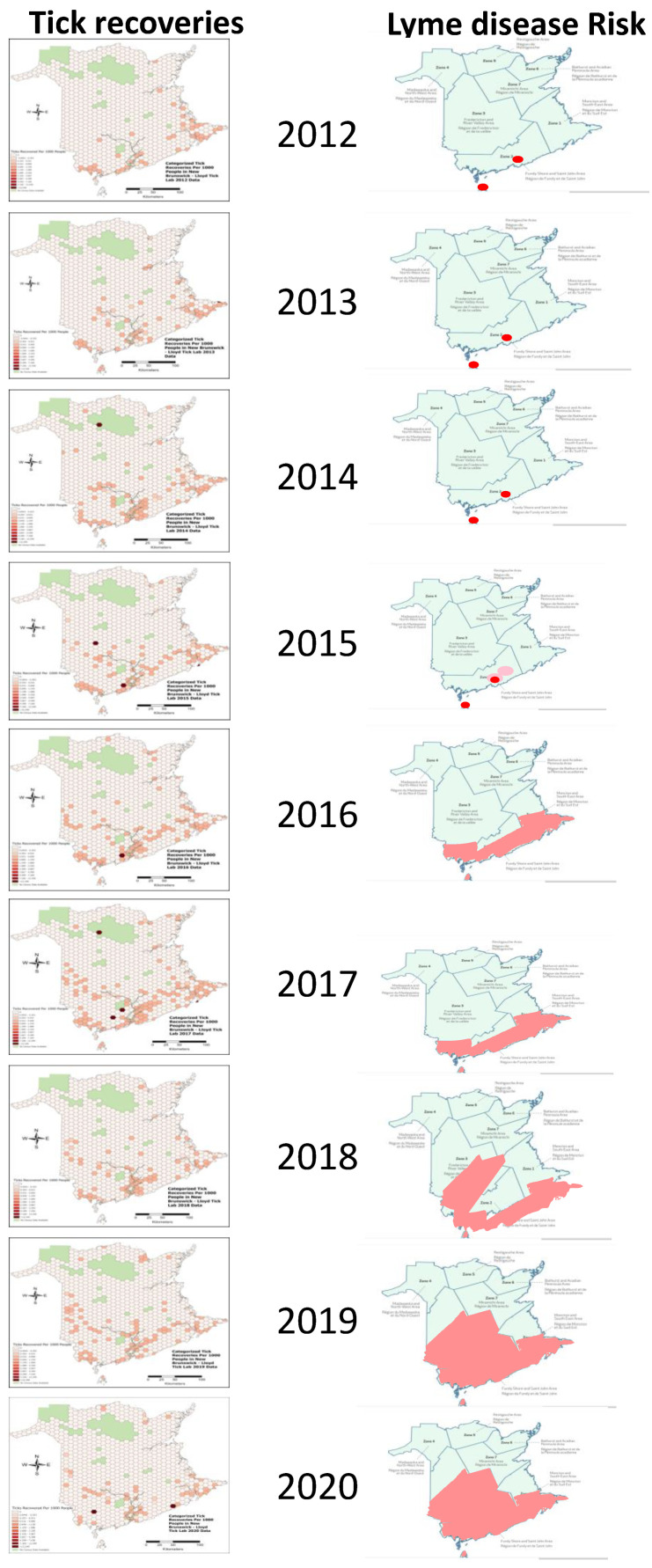
Comparison between tick recovery locations and abundance and public health Ld risk maps for New Brunswick from 2012 to 2020. Images on the left show *Ixodes scapularis* recoveries mapped using a fine-scale hexagon grid map. Recoveries per 1000 people with a decile colour scale applied to the upper 10th percentile of hexagon-level recovery densities; hexagon cells with no recoveries were grouped into a separate category, those with no census data are shown in green. Images on the right are public health Ld risk regions re-drawn from public health risk maps [40,41,48]. In the early maps, red dots indicate known endemic areas, in later years pink indicates broader risk areas. Risk areas may have been added between 2012 and 2015 but maps are not publicly available.

This study was promoted throughout New Brunswick so tick recoveries within the province can be assumed to generally reflect tick abundance. In New Brunswick, *I. scapularis* was collected throughout the province, but mainly from the southern and coastal regions, which include the major population centers of Saint John, Fredericton, and Moncton (Table 3 and Figure 2). At the start of the surveillance period, three clusters of tick recovery were evident in the regions of St. Stephen, Saint John, and Westmorland County (Figure 2). These clusters of increased tick recovery per capita persisted and grew in geographic distribution through the nine-year period. Three major trends were observed over time: growing tick recoveries per capita inland up the Saint John River and beyond, development of growing tick recoveries on a line between Saint John and Westmorland counties and increased recoveries in the north and northeast. Although climate prediction models do not predict that northern New Brunswick would have a climate conducive to tick population establishment [36], when normalized for human population, a key limiting factor in citizen science studies, *I. scapularis* recoveries from the Miramichi watershed, along the Acadian peninsula and along the Chaleur Bay shows appreciable and increasing numbers of tick recoveries, particularly after 2016 (Figure 2). 

Comparison of tick recovery numbers between provinces is problematic due to different recruitment efforts. Although promoted only in New Brunswick, a substantial number of ticks were submitted from the neighboring provinces of Nova Scotia and Prince Edward Island. In Nova Scotia, ticks were submitted from throughout the mainland, but most were submitted from regions close to New Brunswick, due to the location of the university where the study took place, at the New Brunswick-Nova Scotia border. Fewer ticks were submitted from Prince Edward Island, but Foley-Eby et al. [47] reported ticks encountered throughout the province and in highest numbers in the parts of the province nearest to New Brunswick and Nova Scotia. 

**Figure 3:**
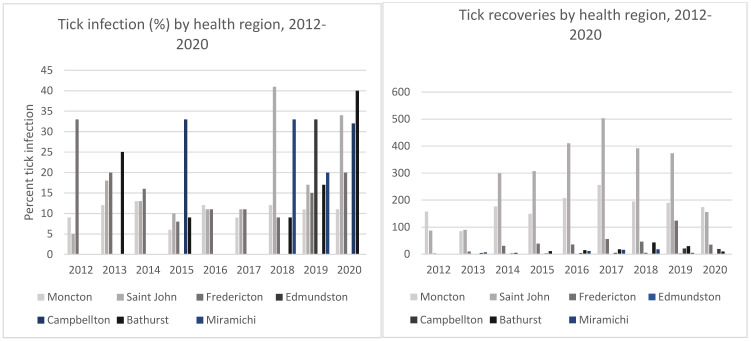
*Borrelia burgdorferi* infection prevalence in *I. scapularis* (left) and *I. scapularis* recoveries (right) across New Brunswick health regions from 2012–2020.

Infection prevalence is not affected by any differential recruitment efforts so it can be compared between provinces (Table 2) and within New Brunswick for which finer-scale geographic tick infection data was collected (Table 3). For all years combined (2012–2020), the province-wide *B. burgdorferi* infection prevalence in *I. scapularis* was 14.1% in New Brunswick (636/4506), and 20.9% in Nova Scotia (172/832) (Table 2). In this study 9.1% (8/88) of ticks from Prince Edward Island infected which is comparable with the 10.3% (37/360) infection prevalence in *I. scapularis* collected from Prince Edward Island from 2016–2017 [47]. For New Brunswick, the province-wide infection percentages generally increased over the nine-year study period, whereas in Nova Scotia it remained relatively stable (Table 2). In New Brunswick, all health district regions showed increased tick recoveries and a trend of increased percentages of *Borrelia*-infected ticks over the course of the study (Table 3, Figure 3). The more northern health districts submitted fewer ticks, but tick recoveries and *Borrelia* infection prevalence did increase over the course of the study (Table 3, Figure 3). 

### 2.3. Borrelia burgdorferi Infection Prevalence

All ticks, regardless of species, were tested for the presence of *B. burgdorferi* (Table 2). The majority of *B. burgdorferi*-infected ticks were *I. scapularis*, as expected. Species other than *I. scapularis* had a low percentage of *Borrelia*-infected ticks, 3.4% for *I. cookei* (24/ 705) and 1.9% for *D. variabilis* (12/619) between 2012 and 2020 (Table 2). 

The host did not have a significant effect on *B. burgdorferi* infection of *I. scapularis* in this collection (Table 4); averaged over the study period, the percent of infection in dogs, cats, humans, other hosts and unknown hosts was 13.3 (n = 3622), 15.6 (n = 817), 17.9 (n = 730), 15.7 (n = 41) and 22.7 (n = 146), respectively. The percent of *I. scapularis* infected with *B. burgdorferi* collected from dogs, cats or humans, averaged across years of collection assessed by one-way ANOVA, was not significantly different at α < 0.05. (*f*-ratio, 1.40267, *p* = 0.265387). 

The incidence of *B. burgdorferi* infection in *I. scapularis* did differ between life stages (Table 5). No larvae were found infected (n = 33), in contrast to adults and nymphs; *B. burgdorferi* was detected in 14.8% of both tick sex adults (n = 5140) and nymphs (n = 215). There was no statistically significant difference between infection frequency in adults and nymphs at α = 0.05 (*t*-test, averaged across years 2012–2020, *p* = 1). 

The incidence of *B. burgdorferi* infection also did not differ by engorgement status. Averaged across years 2012–2020, *B. burgdorferi* was detected in 15.0 (n = 367), 15.1 (n = 3101) and 14.4 (n = 1958) percent of non-engorged, engorged and highly engorged ticks, respectively (Table 6, by one-way ANOVA, *p* = 0.985543). 

In New Brunswick, prior to 2015, there were two known tick endemic areas defined by active surveillance (Figure 2). Five suspected areas, all in the Saint John region, had been added by 2015 (Figure 2). The Moncton and Fredericton regions, the two other major population centers in the province, were not considered to be Ld risk areas until 2016 and 2018, respectively. To assess the correlation between tick population densities and *B. burgdorferi* infection prevalence in a region and the designation of that region as a tick endemic/Ld risk areas, tick recoveries and *B. burgdorferi* infection prevalence were compared between designated endemic/risk areas and those that were not designated as endemic/risk areas. One-way ANOVA analysis, showed that *I. scapularis* recoveries, averaged over the years 2012–2016, which are the years prior to when the risk areas were considered to expand, was indeed significantly different between the regions harboring the three major population centers at α = 0.05 (*f*-ratio value 9.10515, *p* = 0.002577). A post hoc t-test showed that this difference was due to lower tick recoveries from the Fredericton region. However, the difference in recoveries between the St. John region, with known endemic sites, and the Moncton region without known endemic sites, was not significantly different (*t*-value −1.5286, *p* = 0.157357) indicating abundant tick recoveries in the Moncton region prior to its designation as a risk area. Because tick infection frequencies are thought to be highest in endemic regions, the non-endemic Moncton and Fredericton regions would be expected to have lower tick infection frequencies, relative to the St. John region with known established tick populations, if they did not harbor endemic regions. However, one-way ANOVA analysis, showed that the percentage of infected *I. scapularis* from the St. John, Moncton and Fredericton regions, averaged over the years prior to designation of the Moncton and Fredericton regions as risk areas (2012–2016), were not significantly different at α = 0.05 (*f*-ratio value 1.81255, *p*-value 0.205187). This result suggests that despite the apparent great number of ticks encountered and donated in the St. John region, the risk of *B. burgdorferi* infection existed in the Moncton and Fredericton regions during this time period, prior to their designation as Ld risk areas.

## 3. Discussion

Surveillance of ticks and their pathogens provides information that can aid in the management of tick-vectored diseases in human and veterinary medicine. The importance of such research is emphasized by studies from the United States, Canada and Europe showing that the health costs of Ld, both during and after treatment, are appreciable [49,50,51,52]. In this study, we used community-supported passive tick surveillance to monitor the presence of ticks and their *Borrelia burgdorferi* infection status in Nova Scotia, a province with widely distributed tick populations; New Brunswick, a province with establishing tick populations, and Prince Edward Island, a province thought not to have established tick populations. The community-supported citizen science approach allowed not only extensive tick recoveries over wide geographic areas but also promotes community tick awareness. This study, complemented by a targeted study on Prince Edward Island [47], received abundant tick donations of multiple tick species, from a variety of hosts, from all three Maritime provinces. The tick species most often recovered in this study were the generalist feeders, *I. scapularis* (Say, 1821), *I. cookei* (Packard, 1869), and *D. variabilis* (Say, 1821). These results are consistent with other passive surveillance studies, a review of which is provided in Table 7. *B. burgdorferi* infection prevalence for *I. scapularis* in this study was 11.3% in New Brunswick, 20.9% in Nova Scotia and Foley-Eby et al. [47] found 10.3% *I. scapularis* from Prince Edward Island infected in 2016–2017. The infection prevalence found here for Nova Scotia is slightly higher than reported by Ogden et al. [53] and Dibernardo et al. [44]; however, this work provides a larger sample size with better temporal and geographic resolution. 

### 3.1. Borrelia burgdorferi and Other Borrelia Species

Ticks acquire *Borrelia burgdorferi* infections by feeding from infected hosts. While some *Borrelia* species such as *Borrelia miyamotoi* are capable of vertical transmission from an infected female into her eggs, this has not been found for *B. burgdorferi*, so larvae would not be expected to be infected with *B. burgdorferi* until they had fed from an infected host [54,55,56]. This is consistent with our results, although finding of infected larvae is not impossible; larvae can become infected after feeding from an infected host and might be collected prior to molting. *B. burgdorferi* is efficiently retained during tick molts so nymphs and adults, as well as engorged versus non-engorged ticks, would have additional opportunities to acquire infection with each subsequent blood meal. As a result, in ticks collected from the field and so likely feeding from wildlife reservoirs, one would expect to see higher infection prevalence in adults than in the nymphal stage, as well as in engorged vs. non-engorged ticks. In this study, however, ticks were collected from humans and well-cared for companion animals unlikely to be infected prior to the tick feeding so acquiring infection during this terminal feeding is unlikely. This presumably explains the similar infection prevalence of adult and nymphal and engorged versus non-engorged ticks found here. 

Each of the major tick species recovered from humans and companion animals in this study can transmit multiple pathogens causing serious disease, although *B. burgdorferi,* being the most prevalent, is the pathogen of greatest public health concern [1]. The majority of *B. burgdorferi*-infected ticks found in this study were *I. scapularis*, as expected given that this species is the primary vector in eastern Canada [6]. However, a low incidence of infection was reproducibly found in ticks of other species; barring the possibility of experimental error, 6% of *I. cookei* and 1.9% of *D. variabilis* were found to be positive for *Borrelia.* Ticks acquire *B. burgdorferi* by feeding, so if these ticks had fed from an infected host, they might become infected. The acquisition of *Borrelia* by some of the *I. cookei* and *D. variabilis* ticks in this study is thus not overly surprising and consistent with previous studies [57,58,59]. Such infection would not necessarily pose a significant health risk; if the ticks did not efficiently retain the bacteria this would limit transmission to another host. *I. cookei* is considered to pose much less of a human health risk than *I. scapularis* for this reason [15]. However, there is a case report of *I. cookei-*transmitted human infection that shows that some risk of transmission exists [14]. An additional layer of complexity is added by the subsequent finding that some of these ticks were hybrids between *I. scapularis* and *I. cookei* [60]. The vectoral potential of such hybrids is unknown. In contrast, *D. variabilis* is considered an incompetent vector as it does not retain *Borrelia* [58,61,62,63,64,65], however, transmission in rare cases of refeeding cannot be excluded. Both *D. variabilis* and *I. cookei* can transmit pathogens other than *Borrelia* so discounting the health risk posed by these ticks is unwise and, ideally, surveillance should include all tick species that readily feed from humans and the most common pathogens that they transmit. 

In addition to the most common species of *Borrelia* in this region, *B. burgdorferi*, ticks can carry other *Borrelia* strains and species. Crowder et al. [66] reported that 39% of adult *I. scapularis* were infected with at least 2 strains of *B. burgdorferi* sensu stricto. Genospecies other than *B. burgdorferi* sensu stricto have been reported at a low prevalence in Canada, including *B. miyamotoi, B. garinii*, and *B. bissettii* [44,67]. Some of the ticks collected from the Canadian Maritime provinces included in this study were infected with *B. bissettii* as well as *B. burgdorferi* [68] and as the flow of *Borrelia* genospecies is inter-continental, Eurasian genospecies have and will continue to appear in North America. Infection with different *Borrelia* species is associated with varying disease manifestations and are poorly or not detected by the two-tiered serological diagnostic algorithm [69,70]. Thus, the biodiversity of *Borrelia* in Canada is of both theoretical and practical importance. 

The number of tick donations between provinces can only cautiously be used as a measure of the risk from tick-vectored diseases because of the different recruitment effort in each province. However, the *Borrelia* infection prevalence is not influenced by recruitment effects, so that metric is a meaningful indication of risk. In Nova Scotia, tick recoveries were robust and the infection prevalence remained around 20% throughout the study, consistent with the multiple and expanding known established tick populations during this time [39]. The New Brunswick province-wide *B. burgdorferi* infection prevalence was 8% (n = 249) in 2012. From 2012- 2017, the *Borrelia* infection prevalence increased to approximately 10%, with the exception of 2013 where it was 22% (n = 323). After 2018, it increased to approximately 20%. Fluctuation in the percentage of ticks infected may be explained by simple annual climactic fluctuations or by sampling error, although recoveries were plentiful. An alternate possibility is that sampling spanned the peak of *Borrelia* infection that occurs concurrent with tick population establishment. A decrease in *Borrelia* infection prevalence as reservoir hosts become saturated or resistant coupled with increasing number of ticks collected has been postulated to be an early signal of the transition from adventitious ticks to established tick populations [71]. Interestingly, the infection prevalence for Prince Edward Island was also 10% [47] and has been preceded by steadily increasing tick recoveries and reports of higher tick infection prevalence documented through a collaboration between the veterinary community on the Island and Public Health Agency of Canada researchers [72], raising the possibility that small local tick populations might be established on the Island producing localized cryptic endemic locations. 

### 3.2. Tick Surveillance

The risk of a person or animal being infected with *Borrelia,* or other tick-vectored pathogen, is a function of three events; the chance of encountering a tick, the chance of that tick being infected with *Borrelia* and the chance of that tick feeding long enough to transmit infection [73]. The number of ticks in a given region can be determined by either active or passive surveillance, with the latter being more sensitive [34]. In the New Brunswick context, this is demonstrated by comparing the results of active and passive tick surveillance. Gabriele-Rivet et al. [74] and Lewis et al. [37] reported recovery of 5 *I. scapularis* from 159 sites and 9 *I. scapularis* from 66 sites in 2014, respectively, by active surveillance, whereas the passive surveillance results reported here recovered 744 ticks, 582 of them *I. scapularis*, that year. Figure 2 shows that the early public health risk maps, based on active surveillance, reflect the relative tick numbers recovered by passive surveillance, but reduced sensitivity of this approach is apparent in the smaller geographic span of risk areas. 

Passive tick surveillance has been criticized as being too sensitive as it detects both adventitious ticks and established populations [34]. Although the sensitivity of passive surveillance can be artificially reduced by filtering the data for subsets of ticks such immature stages, males, overwintering adults so that it can match the biological filtering inherent in active tick surveillance, the assumption that adventitious ticks can be disregarded in risk assessment is problematic. While lower in density than ticks from local breeding populations, adventitious ticks do tend to come from established endemic populations, where the chance of a given tick being *Borrelia*-infected is high [71]. Without *Borrelia* being established in the local wildlife populations, the initial tick populations seeded from adventitious ticks will be less likely to be infected [71]. However, along migratory routes, episodic introduction of adventitious ticks over time could introduce *Borrelia* into the wildlife population in advance of the growth of substantial local tick populations. While not surprising in a largely forested and rural province, this suggests that particularly for coastal and inland riparian regions, risk modeling based on tick density alone may be inappropriate. This concern is supported by our finding that the incidence of *Borrelia* infection in these ticks did not differ significantly between areas within and external to the designated Ld risk areas for several years prior to their designation as Ld risk areas. Ultimately, the preferred method of tick surveillance is one that best captures the risk to people and animals in a region and the passive surveillance tick recovery described here are consistent with canine [75], bovine [76] and equine [77] surveillance initiatives. Canine sentinel studies, that capture the risk from both adventitious ticks and established tick populations, are recognized as important tools in monitoring and predicting regions where individuals are at heightened risk of contracting tick-vectored diseases [78].

### 3.3. Lyme Disease Risk Predictions

For preventative messaging, the risk predictions should either anticipate risk or be concurrent with risk. In this case, tick presence, abundance, the nature and prevalence of infection should be communicated to the public in a timely manner to promote strategies that reduce tick encounters and reduce the duration of tick feeding. While the saltatory and fluid seeding of adventitious ticks is difficult to model, modeling climate and biotic factors that promote establishment of new tick populations can be used to provide predictive power for public health messaging [36,46,79,80]. Tick establishment is driven by climate conditions, geography and the movement of animal hosts [36,46,81,82,83]. The similarity of the tick recoveries over time and the predicted tick occurrence, as shown in Figure 2, support the predictive power of the occupancy model for New Brunswick, which was constructed using IPCC climate predictions for the 2020’s and based on adult tick recoveries from 2014–2016 [36]. Tick populations were found expanding from the south of New Brunswick, along the coastline and upriver valleys in central New Brunswick, as predicted by the model. The upriver increase along the Saint John River and increased spread between Saint John and Westmorland counties are also the areas of highest probability in the 2020’s occupancy model [36]. This indicates that forward prediction of trends is accurate, at least in the short term, even at small scale and in complex geographies. However, no climate prediction maps predicted the increased tick recoveries along the northeast coastal regions, showing that tick surveillance is still needed to accurately detect tick presence and the attendant risk of tick-borne disease.

In New Brunswick, a province with establishing tick populations, the public health risk maps based on active tick surveillance have not and do not fully capture tick exposure, as can be seen by comparing actual tick recoveries and public health risk maps (Figure 2). Prior to 2015, risk was indicated as being confined to distinct endemic areas, despite substantial tick recoveries over a much broader area. Results of passive tick surveillance conducted in 2016 by public health agencies, published in June 2017, produced findings similar to those documented here; extensive tick recoveries were obtained in the southern third of province including St. John, Moncton and Fredericton areas [84]. This information may have led to expansion of the Ld risk areas, and generalization of endemic areas to broader “risk areas”, which along with the statement that ticks can be encountered anywhere, improves risk messaging to the public. Nevertheless, the officially defined risk areas lag behind substantial tick recoveries by at least 4 years, were infrequently and irregularly updated and still do not encompass coastal areas. New Brunswick is a province with establishing tick populations so the spread of tick populations may be more dynamic than other provinces in which tick populations are either already broadly present, such as Nova Scotia, or thought to be not yet established, as in Prince Edward Island. Nevertheless, endemic areas can expand and the rate of infection increase and adventitious ticks also pose a risk so that timely messaging on tick encounters is necessary to reinforce “tick aware” behaviors regardless of a region’s current tick status. Because climate models do not fully capture tick encounters, as we have shown here, tick surveillance will continue to be necessary. This surveillance must be sensitive, which effectively means passive surveillance with public participation, coupled to rapid communication of surveillance results to the public. Annually updated reports from this study have been available online [85,86]. Even more rapid tick encounter information can be provided by online platforms with real time reporting. Such online platforms have shown great success in accomplishing this task in other countries [87,88] and, in Canada, eTick [89] is a dedicated online platform that allows the public to access tick recoveries in real-time. 

**Table 7:** Summary of studies on *Ixodes scapularis* and *Borrelia burgdorferi* in Canada, 2000–2016.

Reference	Province(s) Studied	Tick Collection Method(s)	Number of *I. scapularis* Collected	*Borrelia* Infection Rate (If Any)
Banerjee et al., 2000 [43]	ON	Passive surveillance (dogs) (1997–1999)	139	2% (n = 121) by culture confirmed by monoclonal antibodies and PCR 6% (n = 121) by PCR
Morshed et al., 2003 [90]	ON	Flagging Trapping small mammals (1999–2000)	263 199	14% of tick pools ^1^ (n = 86 pools) by PCR 40% of white-footed mice (n = 15) by PCR
Scott et al., 2004 [91]	ON	Flagging Trapping small mammals (2001–2002)	254 59	45% of cultured pools ^2^ (n = 53) by PCR 25% of white-footed mice (n = 4) by culturing confirmed with PCR
Ogden et al., 2006a [53]	SK, MB, ON, QC, NB, NS, PEI, and NL	Passive surveillance (1996–2003)	1816	10% (n = 349) in MB 11% (n = 45) in ON 13% (n = 984) in QC 16% (n = 151) in NB 15% (n = 86) in NS 11% (n = 180) in PE I19% (n = 21) in NL 13% (n = 1816) in Canada, all by PCR
Scott et al., 2007 [92]	ON	Flagging (2005–2006)	46	67% of pools ^3^ (n = 15 tick pools) by culture confirmed with PCR
Ogden et al., 2008 [24]	ON, QC, and NS	Birds capturing (2005–2006)	263	15% (n = 205 nymphs) and 0% (n = 53 larvae) in ON No tick was recovered from QC 25% (n = 4 nymphs) in NS, all by PCR
Scott and Durden, 2009 [93]	ON	Bird capturing	7	43% (n = 7) by culture and confirmed with PCR
Ogden et al., 2010 [71]	QC	Flagging and rodent capture (2007–2008) Passive surveillance (1996–2004)	2259 for active surveillance For passive surveillance, numbers are not given but submission per year is shown in their Figure 1	1% (n = 1169) by serological analysis of rodents 1.8–3.3% (n = 675) by PCR of seropositive rodents and ticks; 11 ticks from 1 rodent were pooled
Bouchard et al., 2011 [94]	QC	Rodent trapping (2007–2008)	855	5% (n = 848) of ticks by PCR 1% of rodents were seropositive (n = 887) by immunofluorescence, ELISA, and Western blot
Krakowetz et al., 2011 [95]	MB, ON, and NS	Drag sampling	153	No infection rates provided
Dibernardo et al., 2014 [44]	AB, MB, ON, QC, NB, NS, PEI, and NL	Passive surveillance (2012)	4938	14% (n = 87) in AB 9% (n = 170) in MB 16% (n = 2591) in ON 14% (n = 1479) in QC 7% (n = 366) in NB 12% (n = 34) in NS 10% (n = 178) in PEI 27% (n = 33) in NL All by PCR
Nelder et al., 2014 [45]	ON	Passive surveillance (2008–2012)	7842	15% of pools ^4^ (n = 6046 tick pools) by PCR
Ogden et al., 2014 [96]	AB, SK, MB, QC, ON, NB, NS, PEI, and NL	Drag sampling (2008–2013) and trapping of small mammals Rodent capturing (2007 and May-Oct.2008) Passive surveillance (2004–2012)	Not provided in the report but a map of sites where at least one *I. scapularis* was found is given Rodent results are presented in the article by Bouchard et al., 2011 221	No infection rates provided in this report but ticks were tested at the National Microbiology Laboratory
Simon et al., 2014 [97]	QC	Drag sampling Trapping small mammals	1417 (total for both collection methods)	14% (n = 311) of ticks from dragging, by PCR 1% of ticks from mammals, by PCR
Werden et al., 2014 [98]	ON	Drag sampling (2009–2010) Trapping small mammals (2009–2010)	1354 (total for both collection methods)	Infection rate ranged from 12 to 30% (n = 1354) between sites by PCR
Gabriele-Rivet et al., 2015 [74]	NB	Drag sampling (2014)	5	25% (n = 4) by PCR
Scott et al., 2016 [99]	ON	Flagging	29	41% (n = 29) by PCR

ON: Ontario, SK: Saskatchewan, MB: Manitoba, QC: Quebec, NB: New Brunswick, NS: Nova Scotia, PEI: Prince Edward Island, NL: Newfoundland and Labrador. ^1^ In this study, ticks were tested in pools of up to five adults (normally 3) or 28 larvae. ^2^ In this study, ticks were tested in pools of up to five adults (normally 3) or 7 larvae. ^3^ This study did not discuss the pooling arrangement. ^4^ Pools from this study groups ticks from the same submission together.

## 4. Conclusions 

Ticks are vectors of a number of serious diseases, including Ld. The need for effective and sensitive surveillance needs to be matched by timely communication of risk estimates to the public, recreational and occupational users of outdoor areas, and the veterinary and human healthcare communities. This study provides the largest and highest density information on ticks in the Canadian Maritimes to date and the results have been used to inform residents as well as veterinary and human medical professionals by providing tick identification and infection status of submitted ticks. In addition, the ticks submitted through this study have been used as a national resource for health researchers. This illustrates the intentional integration and respectful partnering with the public and, in particular, those who have increased occupational and recreational exposure to ticks. The results presented here on the value and limitations of tick surveillance demonstrate the need for publicly accessible and integrated information to support tick bite prevention, as well as diagnosis and treatment of tick-vectored diseases. 

## 5. Materials and Methods 

### 5.1. Tick Collection, Identification, and Photography 

Ticks were collected by passive surveillance from veterinarians and the general public across the Maritime provinces between 2012 and 2020 (Animal care protocol 101971). Tick donors filled out a submission form, indicating the tick host, whether tick was attached or not, geographic location of encounter, date of tick collection, and travel within the last 2 weeks, if any. Community-based tick collection method used in this study had the advantages and limitations typically associated with this type of collection process. While efficient, there was a recruitment bias introduced by uneven public awareness of the tick surveillance program. At the initiation of the program, notices were sent to all veterinary clinics across the province of New Brunswick and there was extensive province-wide media coverage. Nevertheless, in the first year of the study the region in the immediate vicinity of the university contributed 12% of the submitted ticks from New Brunswick, from only 3 veterinary clinics (corresponding to 4% of the total number of veterinary clinics in the province). By 2017, the Sackville area contributed only 6% of the total ticks received, suggesting that the regional bias had largely resolved. There was no active promotion of the project in Nova Scotia or Prince Edward Island. However, over the course of the study, awareness of the program expanded, in large part by word of mouth, media coverage, through the lab website and through veterinary networks, subsequently decreasing the extent of the regional bias.

Upon receipt, tick specimens were morphologically identified to species, developmental stage, sex, and engorgement status based on standard keys [100]. Ticks were photographed and laterally bisected, with half archived at −20 °C for future study and the other used for molecular testing. Ticks were tested individually and were not pooled. 

### 5.2. DNA Extraction

DNA extraction was performed in a LabGard Class II A2 biological safety cabinet using Aquagenomic solution (MultiTarget Pharmaceuticals) following a modified version of the manufacturer’s tissue protocol, as described by Wills et al. [101]. Briefly, ticks were homogenized using an Eppendorf pestle (Diamed) in 50–200 µL of Aquagenomic solution, depending on the tick’s size and engorgement. The samples were then incubated in a water bath at 60 °C for 45 min, vortexed, and centrifuged for four minutes at 13,300 rpm in a desktop microcentrifuge (Spectrafuge 24D Digital Microcentrifuge). The supernatant was transferred into another Eppendorf tube containing 50–200 µL of isopropanol, mixed by inversion, and centrifuged as before. The supernatant was decanted and the DNA pellet rinsed with 50 µL of 70% ethanol. Excess ethanol was pipetted out and the pellet was air-dried for 15 min at room temperature and 50 µL of 1 mM Tris pH 7.0 was added. DNA samples were incubated in a water bath for one hour at 60 °C to resuspend the DNA, and samples were stored at −20 °C for further analysis. 

### 5.3. Nested Polymerase Chain Reaction

Nested PCR (nPCR) was performed to detect *Borrelia* DNA. From 2012–2017, two *B. burgdorferi* genes, *Outer surface protein* A (*OspA*), a plasmid-encoded gene, and *Flagellin B* (*FlagB*), a chromosomal gene, were assayed. *Borrelia*-positive ticks were defined as those with amplification of both *Borrelia* genes. From 2018 to 2020, the *23S ribosomal RNA* (*rRNA*) gene was assayed. The outer primers amplify the *23S ribosomal RNA* (*rRNA*) gene of all *Borrelia* spp. [44] and the inner set is specific for *B. burgdorferi* [102]. Reactions were set up in a PCR cabinet (Misonix) pre-sterilized with UV light and 70% ethanol. The reaction mixture for the first round consisted of 12.5 µL of GoTaq Green Master Mix 2X (Promega), 8.5 µL sterile nuclease-free water, 1 µL each of 10 µM forward and reverse outer primers (Table 8), and 2 µL of the extracted DNA or, for negative controls, sterile nuclease-free water. The second round was carried out as the first except for the use of inner primers instead of outer primers (Table 8) and 2 µL of first round PCR product in lieu of the extracted DNA or water for negative controls. The thermal cycler program was as follows: 95 °C for five minutes; 40 cycles of 95°C for 15 s, annealing temperature for 30 s, 72 °C for 45 s; 72 °C for five minutes; and hold at 4 °C. The annealing temperature for each primer is listed in Table 8. Amplicons were visualized following electrophoresis in 1.2% agarose gel in SB buffer (0.02 M NaOH, 0.075 M boric acid, pH 8) for one hour at 107 V. Gels were imaged using a BioRad Fluor-S™ MultiImager with the Quantity One^®^ 1-D analysis software version 4.5.2. While nPCR is considered the most sensitive type of PCR analysis, it is also subject to false positives from contamination [103]. To guard against contamination, all stages of DNA handling (DNA isolation from ticks, PCR reaction preparation, gel electrophoresis) were performed in different rooms with air flows isolated from each other. Additionally, two negative controls were used, one prepared before opening the Eppendorf tube with DNA (before control) and one after (after control). The “before” negative control indicates contamination of reagents while the “after” negative control indicates possible aerosol contamination. Suspect results were discarded and repeated. 

### 5.4. Geomapping Tick Recoveries

Tick recoveries from outside of New Brunswick, with uncertain encounter locations or “outside of community” travel history in the 2 weeks prior to recovery were removed. Tick recovery locations were geocoded using an online geocoding resource (https://www.geocod.io/) to assign a latitude and longitude based on the community in which the tick was found in cases were detailed encounter location was unavailable. Annual tick recovery maps were created using the geographic information systems (GIS) software, *ArcGIS*, with three layers. The layers were a population census layer obtained from the Center For International Earth Science Information Network-CIESIN-Columbia University, (2018), a point layer that had individual tick recoveries in New Brunswick for the respective year, and a zonal hexagon layer that divided New Brunswick into ~96,010 km tall, 11.55 km wide hexagons [36]. These layers allowed the tick recovery data to be mapped relative to both location and human per capita values. In citizen science initiatives the human population is a key factor in tick recoveries and the tick recovery data much be corrected for this factor. Some regions encompassing provincial parks, military installations, etc., lacked census data. Across all years, ~90% of the calculated per capita values resulted in zero or near zero values arising from a low number of ticks found within the respective zone. Therefore, across all years a categorization method was applied that collapsed all zero and near zero values into a single category and expanded the upper 10% of values for examination and visualization into 11 categories. Therefore, what resulted was a field entitled *tickspercapita2012_cat*, a categorized field which allowed visual trends to be identified in the tick recoveries per capita throughout the eight-year period. 

## Figures and Tables

**Table 1:** Tick donation by species and life stages between 2012 and 2020.

		*I. scapularis*					*I. cookei*					*D. variabilis*					Unknown ^1^					Other Tick Species ^2^					
		Adult Female	Adult Male	Nymph	Larvae	Total *I. scapularis*	Adult Female	Adult Male	Nymph	Larvae	Total *I. cookei*	Adult Female	Adult Male	Nymph	Larvae	Total *D. variabilis*	Adult Female	Adult Male	Nymph	Larvae	Total Unknown	Adult Female	Adult Male	Nymph	Larvae	Total Other Species	Total All Species
2012	Dog	167	8	2	0	177	14	0	0	0	14	3	4	0	0	7	8	1	0	0	9	0	0	0	0	0	207
	Cat	63	4	3	0	70	16	0	0	0	16	0	0	0	0	0	11	2	0	0	13	0	0	0	0	0	99
	Human	13	1	0	20	34	3	0	0	0	3	3	1	0	0	4	2	0	0	0	2	0	0	0	0	0	43
	Other host ^3^	0	0	0	0	0	3	0	2	0	5	0	0	0	0	0	0	0	1	0	1	0	0	0	0	0	6
	Undetermined ^4^	8	2	0	0	10	0	0	0	0	0	0	0	0	0	0	0	0	0	0	0	0	0	0	0	0	10
	Total 2012	251	15	5	20	291	36	0	2	0	38	6	5	0	0	11	21	3	1	0	25	0	0	0	0	0	365
2013	Dog	311	15	4	0	330	23	0	4	6	33	15	4	0	0	19	3	1	0	4	8	2	1	0	0	1	391
	Cat	43	3	2	0	48	48	0	1	0	49	1	0	0	0	1	0	0	0	0	0	0	0	0	0	0	98
	Human	26	1	3	6	36	6	6	0	0	12	17	2	2	5	26	1	0	0	2	3	1	0	0	0	0	77
	Other host ^3^	1	0	0	0	1	0	0	0	0	0	0	0	0	0	0	0	0	0	0	0	0	0	0	0	0	1
	Undetermined ^4^	4	0	1	0	5	0	0	0	0	0	13	0	0	0	13	0	0	0	0	0	0	0	0	0	0	18
	Total 2013	385	19	10	6	420	77	6	5	6	94	46	6	2	5	59	4	1	0	6	11	3	1	0	0	1	585
2014	Dog	426	18	5	0	449	59	2	4	14	79	12	2	0	0	14	22	0	0	6	28	0	0	0	0	0	570
	Cat	71	3	2	0	76	23	0	11	5	39	0	0	0	0	0	6	0	0	0	6	0	0	0	0	0	121
	Human	33	4	5	0	42	1	0	4	0	5	10	6	0	0	16	1	0	0	0	1	0	0	0	0	0	64
	Other host ^3^	5	1	0	0	6	0	0	0	0	0	0	1	0	0	1	0	0	0	0	0	0	0	0	0	0	7
	Undetermined ^4^	8	0	1	0	9	0	0	1	0	1	1	0	0	0	1	0	0	1	0	1	0	0	0	0	0	12
	Total 2014	543	26	13	0	582	83	2	20	19	124	23	9	0	0	32	29	0	1	6	36	0	0	0	0	0	774
2015	Dog	400	9	9	4	422	33	0	16	1	50	38	18	0	0	56	7	0	4	0	11	1	0		0	0	539
	Cat	76	2	12	0	90	19	0	19	0	38	0	0	0	0	0	1	0	1	0	2	0	3	0	0	3	133
	Human	29	3	14	0	46	2	0	4	0	6	16	18	2	0	36	1	1	3	0	5	0	0	0	0	0	93
	Other host ^3^	1	0	0	0	1	0	0	0	0	0	3	2	0	0	5	1	0	3	0	4	0	0	0	0	0	10
	Undetermined ^4^	11	0	2	0	13	0	0	0	0	0	1	1	0	0	2	1	0	0	0	1	0	0	0	0	0	16
	Total 2015	517	14	37	4	572	54	0	39	1	94	58	39	2	0	99	11	1	11	0	23	1	3	0	0	3	791
2016	Dog	520	24	6	0	550	14	0	8	12	34	12	3	1	0	16	14	5	2	0	21	9	1	0	0	1	622
	Cat	132	6	6	1	145	20	0	22	11	53	4	0	0	0	4	4	0	1	3	8	0	0	0	0	0	210
	Human	77	6	8	1	92	2	0	2	0	4	24	13	0	0	37	2	0	0	0	2	0	0	0	0	0	135
	Other host ^3^	7	1	0	0	8	0	0	0	0	0	11	0	0	0	11	1	0	0	0	1	0	0	0	0	0	20
	Undetermined ^4^	18	9	0	0	27	0	0	0	0	0	2	0	0	0	2	0	1	0	0	1	0	0	0	0	0	30
	Total 2016	754	46	20	2	822	36	0	32	23	91	53	16	1	0	70	21	6	3	3	33	9	1	0	0	1	1017
2017	Dog	607	44	5	1	657	7	0	26	7	40	29	13	0	0	42	18	7	8	0	33	4	3	1	0	4	776
	Cat	140	9	10	0	159	10	0	16	0	26	1	0	0	0	1	10	1	2	0	13	0	0	0	0	0	199
	Human	160	7	38	0	205	3	0	6	0	9	27	32	0	1	60	4	2	6	0	12	1	0	1	0	1	287
	Other host ^3^	8	0	2	0	10	0	0	8	1	9	0	0	1	0	1	1	0	0	0	1	2	0	0	0	0	21
	Undetermined ^4^	37	4	1	0	42	0	0	2	0	2	8	5	0	1	14	2	0	0	0	2	0	0	0	0	0	60
	Total 2017	952	64	56	1	1073	20	0	58	8	86	65	50	1	2	118	35	10	16	0	61	7	3	2	0	5	1343
2018	Dog	309	27	2	0	338	19	2	16	5	42	20	13	0	0	33	0	1	0	0	1	0	3	0	0	3	417
	Cat	98	2	17	0	117	20	0	26	1	47	3	0	0	0	3	0	0	0	0	0	2	0	0	0	0	167
	Human	71	12	18	0	101	5	0	11	0	16	41	22	0	1	64	0	0	0	0	0	15	0	2	0	2	183
	Other host ^3^	5	0	0	0	5	1	0	0	0	1	0	0	0	0	0	0	0	0	0	0	0	1	0	0	1	7
	Undetermined ^4^	11	0	0	0	11	0	0	0	0	0	3	0	8	0	11	1	0	0	0	1	0	0	0	0	0	23
	Total 2018	494	41	37	0	572	45	2	53	6	106	67	35	8	1	111	1	1	0	0	2	17	4	2	0	6	797
2019	Dog	471	29	2	0	502	24	0	9	2	35	28	15	1	0	44	0	1	0	0	1	2	1	1	0	2	584
	Cat	115	2	2	0	119	14	0	21	4	39	0	2	0	0	2	0	1	0	0	1	0	0	0	0	0	161
	Human	217	7	31	0	255	4	0	7	0	11	39	32	2	0	73	0	0	1	1	2	2	0	3	0	3	344
	Other host ^3^	4	0	0	0	4	3	0	0	0	3	1	1	0	0	2	0	0	0	0	0	2	0	0	0	0	9
	Undetermined ^4^	50	7	2	0	59	0	0	2	0	2	8	5	0	1	14	2	0	0	0	2	0	0	0	0	0	77
	Total 2019	857	45	37	0	939	45	0	39	6	90	76	55	3	1	135	2	2	1	1	6	6	1	4	0	5	1175
2020	Dog	243	23	0	0	266	15	0	6	0	21	8	4	0	0	12	2	0	5	0	7	0	1	0	0	1	307
	Cat	23	1	2	0	26	1	0	8	1	10	0	0	0	0	0	2	0	5	0	7	0	0	0	0	0	43
	Human	46	6	2	0	54	1	0	3	0	4	6	1	0	1	8	0	0	0	0	0	1	0	0	0	0	66
	Other host ^3^	4	0	0	0	4	0	0	0	0	0	0	0	0	0	0	0	0	0	0	0	0	0	0	0	0	4
	Undetermined ^4^	9	0	0	0	9	1	0	1	1	3	0	0	0	0	0	0	0	0	0	0	0	0	0	0	0	12
	Total 2020	325	30	4	0	359	18	0	18	2	38	14	5	0	1	20	4	0	10	0	14	1	1	0	0	1	432

^1^ The “Unknown tick species” category, 2.9% of all submissions (211/7323), includes specimens too badly damaged for species determination.^2^ The “Other tick species” category includes seabird ticks, rabbit ticks, brown dog ticks and mouse ticks. ^3^ The “Other host” category includes horse, cow, pig, seabirds, songbirds, mouse species, shrew, fox, coyote, skunk, groundhog, moose, deer, bear, squirrel, raccoon, vole and hare. ^4^ The “Undetermined host” category includes unfed ticks found outdoors or fully engorged detached ticks.

**Table 2:** *Borrelia burgdorferi* infection by tick species between 2012 and 2020.

Year of Collection	Collection Location	Percentage of Tick Infection (%) and Samples Size (n) by Tick Species:				
		*I. scapularis*	*I. cookei*	*D. variabilis*	Unknown Species ^1^	Other Species ^2^
2012	NB	8 (n = 20/249)	7 (n = 1/14)	11 (n = 1/9)	15 (n = 4/26)	0 (n = 0/0)
	NS	23 (n = 9/39)	25 (n = 1/4)	100 (n = 1/1)	0 (n = 0/1)	0 (n = 0/0)
	PEI	0 (n = 0/0)	0 (n = 0/0)	0 (n = 0/0)	0 (n = 0/0)	0 (n = 0/0)
	Other ^3^	33 (n = 1/3)	0 (n = 0/0)	50 (n = 1/2)	0 (n = 0/0)	0 (n = 0/0)
2013	NB	22 (n = 71/323)	4 (n = 3/84)	0 (n = 0/35)	0 (n = 0/9)	0 (n = 0/1)
	NS	14 (n = 8/58)	0 (n = 0/2)	0 (n = 0/15)	0 (n = 0/2)	0 (n = 0/0)
	PEI	0 (n = 0/0)	0 (n = 0/0)	0 (n = 0/0)	0 (n = 0/0)	0 (n = 0/0)
	Other ^3^	33 (n = 1/3)	0 (n = 0/0)	33 (n = 1/3)	0 (n = 0/0)	0 (n = 0/0)
2014	NB	13 (n = 67/516)	5 (n = 5/109)	7 (n = 1/14)	9 (n = 3/34)	0 (n = 0/0)
	NS	17 (n = 9/53)	13 (n = 1/8)	0 (n = 0/9)	0 (n = 0/2)	0 (n = 0/0)
	PEI	0 (n = 0/0)	0 (n = 0/0)	0 (n = 0/0)	0 (n = 0/8)	0 (n = 0/0)
	Other ^3^	0 (n = 0/0)	0 (n = 0/0)	0 (n = 0/0)	0 (n = 0/1)	0 (n = 0/0)
2015	NB	10 (n = 47/465)	2 (n = 2/90)	0 (n = 0/28)	0 (n = 0/21)	0 (n = 0/3)
	NS	16 (n = 13/82)	0 (n = 0/7)	0 (n = 0/60)	0 (n = 0/0)	0 (n = 0/1)
	PEI	0 (n = 0/0)	0 (n = 0/0)	0 (n = 0/0)	0 (n = 0/0)	0 (n = 0/0)
	Other ^3^	13 (n = 1/8)	0 (n = 0/2)	0 (n = 0/10)	0 (n = 0/3)	0 (n = 0/0)
2016	NB	11 (n = 75/682)	9 (n = 7/76)	4 (n = 1/24)	5 (n = 1/22)	0 (n = 0/10)
	NS	26 (n = 29/113)	0 (n = 0/4)	0 (n = 0/31)	0 (n = 0/3)	0 (n = 0/0)
	PEI	33 (n = 1/3)	0 (n = 0/0)	0 (n = 0/0)	0 (n = 0/1)	0 (n = 0/0)
	Other ^3^	25 (n = 2/8)	0 (n = 0/0)	0 (n = 0/16)	0 (n = 0/14)	0 (n = 0/1)
2017	NB	10 (n = 84/841)	2 (n = 1/66)	0 (n = 0/36)	0 (n = 0/52)	0 (n = 0/12)
	NS	21 (n = 38/179)	0 (n = 0/8)	0 (n = 0/59)	0 (n = 0/9)	0 (n = 0/0)
	PEI	0 (n = 0/1)	0 (n = 0/0)	0 (n = 0/0)	0 (n = 0/0)	0 (n = 0/0)
	Other ^3^	15 (n = 6/41)	0 (n = 0/1)	0 (n = 0/13)	0 (n = 0/7)	0 (n = 0/0)
2018	NB	22 (n = 144/519)	5 (n = 4/88)	5 (n = 2/40)	22 (n = 2/9)	0 (n = 0/24)
	NS	25 (n = 28/112)	0 (n = 0/12)	0 (n = 0/55)	0 (n = 0/0)	0 (n = 0/0)
	PEI	25 (n = 4/16)	0 (n = 0/0)	0 (n = 0/2)	0 (n = 0/0)	100 (n1/1)
	Other ^3^	24 (n = 8/33)	33 (n = 1/3)	0 (n = 0/7)	0 (n = 0/0)	0 (n = 0/0)
2019	NB	17 (n = 103/604)	2 (n = 2/81)	0 (n = 0/39)	7 (n = 1/14)	0 (n = 0/8)
	NS	21 (n = 31/147)	0 (n = 0/8)	2 (n = 2/89)	0 (n = 0/7)	0 (n = 0/0)
	PEI	8 (n = 1/13)	0 (n = 0/0)	0 (n = 0/1)	0 (n = 0/1)	0 (n = 0/0)
	Other ^3^	30 (n = 6/20)	0 (n = 0/1)	0 (n = 0/1)	0 (n = 0/0)	0 (n = 0/0)
2020	NB	18 (n = 55/307)	5 (n = 2/37)	15 (n = 2/13)	19 (n = 3/16)	0 (n = 0/1)
	NS	18 (n = 7/40)	0 (n = 0/0)	0 (n = 0/5)	0 (n = 0/0)	0 (n = 0/0)
	PEI	8 (n = 2/25)	0 (n = 0/0)	0 (n = 0/1)	0 (n = 0/0)	0 (n = 0/0)
	Other ^3^	0 (n = 0/4)	0 (n = 0/0)	0 (n = 0/1)	0 (n = 0/0)	0 (n = 0/1)

^1^ The “Unknown tick species” category includes specimens too badly damaged for species determination. ^2^ The “Other tick species” category includes seabird ticks, rabbit ticks, brown dog ticks and mouse ticks. ^3^ The “Other collection location” category includes ticks from locations other Canadian provinces, the United States, South America, Africa, Asia, Australia and Europe. NA = no counts for that category. Ticks from unknown or multiple possible locations are excluded.

**Table 3:** *Ixodes scapularis* tick recoveries and percent tick infection from each New Brunswick health region between 2012 and 2020.

Year of Collection	Percentage of Tick, All Species, Infection (%) and Sample Size (n) by Health Regions:						
	Moncton	Saint John	Fredericton	Edmundston	Campbellton	Bathurst	Miramichi
2012	9 (n = 14/157)	5 (n = 4/87)	33 (n = 1/3)	0 (n = 0/0)	0 (n = 0/0)	0 (n = 0/2)	0 (n = 0/0)
2013	12 (n = 10/85)	18 (n = 16/90)	20 (n = 2/10)	0 (n = 0/0)	0 (n = 0/1)	25 (n = 1/4)	0 (n = 0/7)
2014	13 (n = 23/176)	13 (n = 39/299)	16 (n = 5/31)	0 (n = 0/2)	0 (n = 0/3)	0 (n = 0/5)	0 (n = 0/0)
2015	6 (n = 9/149)	10 (n = 31/307)	8 (n = 3/39)	0 (n = 0/1)	33 (n = 1/3)	9 (n = 1/11)	0 (n = 0/2)
2016	12 (n = 25/208)	11 (n = 45/410)	11 (n = 4/36)	0 (n = 0/1)	0 (n = 0/4)	0 (n = 0/15)	0 (n = 0/12)
2017	9 (n = 23/256)	11 (n = 55/503)	11 (n = 6/56)	0 (n = 0/2)	0 (n = 0/5)	0 (n = 0/18)	0 (n = 0/16)
2018	12 (n= 23/195)	41 (n = 161/392)	9 (n = 4/46)	0 (n = 0/5)	0 (n = 0/2)	9 (n = 4/43)	33 (n = 6/18)
2019	11 (n = 21/190)	17 (n = 63/373)	15 (n = 19/124)	33 (n = 1/3)	0 (n = 0/21)	17 (n = 5/30)	20 (n = 1/5)
2020	11 (n = 19/174)	34 (n = 53/156)	20 (n = 7/35)	0 (n = 0/1)	32 (n = 6/19)	40 (n = 4/10)	0 (n = 0/1)

**Table 4:** *Borrelia burgdorferi* infection prevalence of *Ixodes scapularis* by host.

Year of Collection	Percentage of Tick Infection (%) and Samples Size (n) by Host:				
	Dog	Cat	Human	Other Hosts ^1^	Unknown Hosts ^2^
2012	9 (n = 16/177)	14 (n = 10/70)	3 (n = 1/34)	50 (n = 1/2)	25 (n = 2/8)
2013	15 (n = 26/172)	10 (n = 3/29)	27 (n = 7/26)	0 (n = 0/1)	33 (n = 1/3)
2014	12 (n = 50/415)	16 (n = 10/64)	16 (n = 4/25)	0 (n = 0/6)	33 (n = 2/6)
2015	10 (n = 42/423)	12 (n = 11/91)	9 (n = 4/46)	0 (n = 0/1)	39 (n = 5/13)
2016	11 (n = 61/550)	14 (n = 20/145)	27 (n = 25/92)	0 (n = 0/9)	6 (n = 1/18)
2017	10 (n = 67/665)	12 (n = 19/161)	17 (n = 36/209)	18 (n = 2/11)	21 (n = 9/42)
2018	20 (n = 85/424)	20 (n = 23/117)	25 (n = 32/129)	0 (n = 0/3)	21 (n = 3/14)
2019	16 (n = 81/509)	23 (n = 26/114)	21 (n = 24/114)	40 (n = 2/5)	16 (n = 5/32)
2020	17 (n = 49/287)	19 (n = 5/26)	16 (n = 9/55)	33 (n = 1/3)	10 (n = 1/10)

^1^ The “Other hosts” category includes horses, groundhogs, coyotes, moose, deer, bears, cows, skunks, raccoons and rabbits. ^2^ The “Unknown hosts” category includes unfed ticks found outdoors not attached to a host or fully engorged detached ticks.

**Table 5:** *Borrelia burgdorferi* infection prevalence of *Ixodes scapularis* by life stage.

Year of Collection	Percentage of Tick Infection (%) and Samples Size (n) by Developmental Stage:			
	Adult	Nymph	Larvae	Unknown
2012	11 (n = 29/266)	40 (n = 2/5)	0 (n = 0/20)	0 (n = 0/0)
2013	17 (n = 37/216)	13 (n = 1/8)	0 (n = 0/6)	0 (n = 0/0)
2014	13 (n = 74/569)	8 (n = 1/12)	0 (n = 0/0)	0 (n = 0/0)
2015	11 (n = 58/531)	8 (n = 3/37)	0 (n = 0/4)	0 (n = 0/3)
2016	14 (n = 112/801)	5 (n = 1/20)	0 (n = 0/2)	0 (n = 0/0)
2017	13 (n = 123/945)	11 (n = 6/56)	0 (n = 0/1)	0 (n = 0/0)
2018	20 (n = 135/675)	20 (n = 6/30)	0 (n = 0/0)	0 (n = 0/0)
2019	17 (n = 129/760)	28 (n = 12/43)	0 (n = 0/0)	0 (n = 0/0)
2020	17 (n = 64/377)	0 (n = 0/4)	0 (n = 0/0)	0 (n = 0/0)

**Table 6:** *Borrelia burgdorferi* infection prevalence of *Ixodes scapularis* by engorgement status.

Year of Collection	Percentage of Tick Infection (%) and Sample Size (n) by Engorgement Status:.		
	Non-Engorged	Engorged	Highly Engorged
2012	8 (n = 3/36)	11 (n = 21/193)	8 (n = 5/63)
2013	40 (n = 8/20)	11 (n = 14/130)	19 (n = 15/80)
2014	4 (n = 1/26)	14 (n = 53/380)	13 (n = 23/176)
2015	10 (n = 2/21)	13 (n = 46/354)	7 (n = 14/200)
2016	13 (n = 6/47)	17 (n = 65/385)	10 (n = 39/389)
2017	20 (n = 13/65)	12 (n = 73/606)	11 (n = 46/417)
2018	33 (n = 1854)	22 (n = 81/368)	17 (n = 44/260)
2019	7 (n = 5/68)	18 (n = 86/477)	26 (n = 60/230)
2020	0 (n = 0/30)	18 (n = 37/208)	19 (n = 27/143)

**Table 8:** Nested primer sets used to detect *Borrelia burgdorferi*.

Year	Primer Name	Target Gene	Sequence (5′-3′)	Annealing Temperature (℃)	Amplicon Size (bp)	Source
2012	OspA out ^1^ R1	*OspA*	GTTAGCAGCCTTGACGAGA	60	272	Ogden et al. 2006 [53] (OspA1b)
	OspA out F1		GATACTAGTGTTTTGCCATC			Ogden et al. 2006 [53] (OspA4b)
	OspA in ^1^ R1	*OspA*	GCGTTTCAGTAGATTTGCCTG	60	214	Ogden et al. 2006 [53] (OspA2b)
	OspA in F1		TCAAGTGTGGTTTGACCTAG			Ogden et al. 2006 [53] (OspA3b)
	FlagB out R1	*FlagB*	AATTGCATACTCAGTACTATTCTTTATAGAT	60	601	Ogden et al. 2006 [53] (fla outer 2)
	FlagB out F1		AAGTAGAAAAAGTCTTAGTAAGAATGAAGGA			Ogden et al. 2006 [53] (fla outer 1)
	FlagB in R1	*FlagB*	GAAGGTGCTGTAGCAGGTGCTGGCTGT	60	390	Ogden et al. 2006 [53] (fla inner 2)
	FlagB in F1		CACATATTCAGATGCAGACAGAGGTTCTA			Ogden et al. 2006 [53] (fla inner 1)
2013	OspA out R4	*OspA*	ACAAGAGCAGACGGAACCAG	60	358	This work
	OspA out F4		CCCCTCTAATTTGGTGCCAT			
	OspA in R4	*OspA*	CACAGGAATTAAAAGCGATGG	60	220	This work
	OspA in F4		AGTGCCTGAATTCCAAGCTG			
	OspA out R3	*OspA*	GTAATTTCAACTGCTGACCCC	60	561	This work
	OspA out F3		TGAAGGCGTAAAAGCTGAC			
	OspA in R3	*OspA*	TTGGTGCCATTTGAGTCGTA	60	330	This work
	OspA in F3		ACTTGAATACACAGGAATTA			
	FlagB out R2	*FlagB*	TGGGGAACTTGATTAGCCTG	60	493	This work
	FlagB out F2		TCATTGCCATTGCAGATTGT			
	FlagB in R2	*FlagB*	TCATTGCCATTGCAGATTGT	60	437	This work
	FlagB in F2		CTTTAAGAGTTCATGTTGGAG			
2014 to 2017	OspA out R2	*OspA*	CAACTGCTGACCCCTCTAAT	55	487	This work
	OspA out F2		CTTGAAGTTTTCAAAGAAGAT			
	OspA in R2	*OspA*	TTGGTGCCATTTGAGTCGTA	58	350	This work
	OspA in F2		ACAAGAGCAGACGGAACCAG			
	FlagB out R3	*FlagB*	GCATCACTTTCAGGGTCTCA	55	503	This work
	FlagB out F3		TGGGGAACTTGATTAGCCTG			
	FlagB in R3	*FlagB*	CTTTAAGAGTTCATGTTGGAG	58	447	This work
	FlagB in F3		TCATTGCCATTGCAGATTGT			
2017	OspA out R2	*OspA*	CAACTGCTGACCCCTCTAAT	55	487	This work
	OspA out F2		CTTGAAGTTTTCAAAGAAGAT			
2018 to 2020	23S out F	*Borrelia* spp. *23S rRNA*	GTATGTTTAGTGAGGGGGGTG	50	587	Dibernardo et al. 2014 [44]
	23S out R		GGATCATAGCTAGGTGGTTAG			
	23S in F	*Borrelia* spp. *23S rRNA*	ATGTATTCCATTGTTTTAATTACG	51	340	Zinck et al. 2021 [102]
	23S in R		GACAAGTATTGTAGCGAGC			

^1^ “out” designates the outer nPCR primer sets and “in” designates inner nPCR primer sets.

## Supplementary Materials

The following are available online at https://www.mdpi.com/article/10.3390/pathogens10101284/s1, Figure S1: Seasonal tick recoveries by year. Figure S2: Seasonal recoveries of *Ixodes scapularis* (**A**), *Ixodes cookei* (**B**) and *Dermacentor variabilis* (**C**) in New Brunswick (NB), Nova Scotia (NS) and Prince Edward Island (PEI). The proportion of the annual tick submissions, per province, is show by month of collection. Insufficient *Ixodes cookei* and *Dermacentor variabilis* were recovered from Prince Edward Island for comparison.
