# Supplementary material for: Monitoring Risk: Tick and Borrelia burgdorferi Public Participatory Surveillance in the Canadian Maritimes, 2012–2020
Source: Pathogens. 2021 Oct 6;10(10):1284. doi: 10.3390/pathogens10101284 (PMC8538556; doi:10.3390/pathogens10101284)
Supplement: Supplementary file 1 [file pathogens-10-01284-s001.zip › pathogens-1381142-supplementary.pdf]

Supplemental Figure S1. Seasonal tick recoveries by year

A

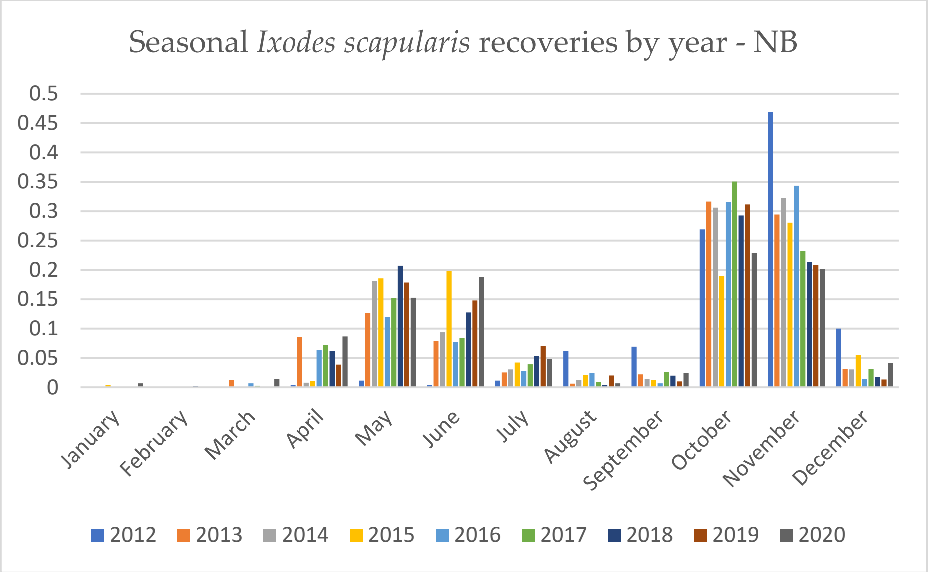

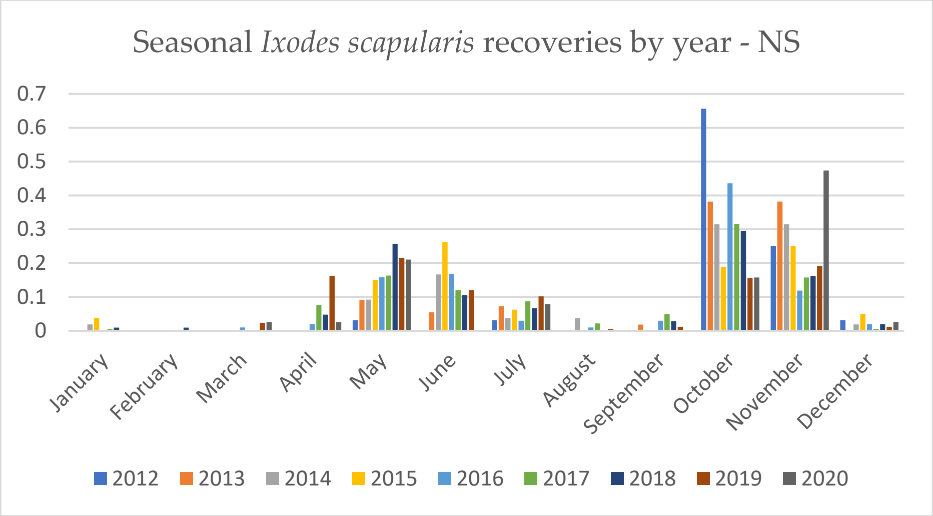

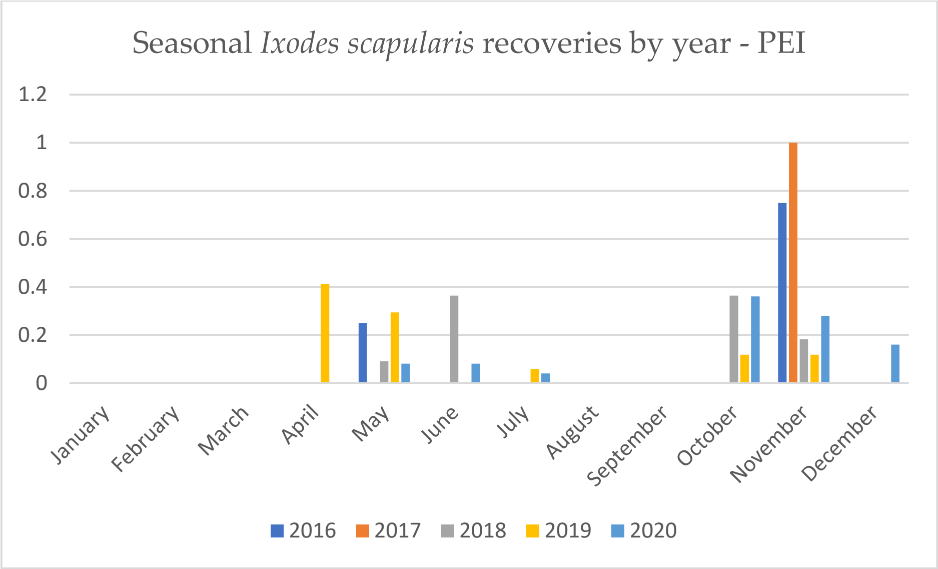
Seasonal *Ixodes scapularis* recoveries by year - PEI
1.2
1
0.8
0.6
0.4
0.2
0
January
February
March
April
May
June
July
August
September
October
November
December
2016
2017
2018
2019
2020

B

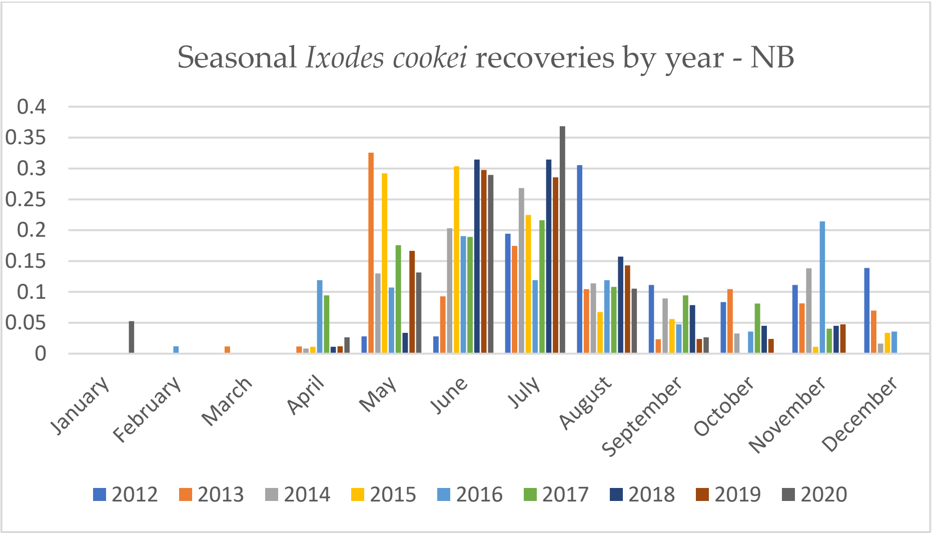
Seasonal *Ixodes cookei* recoveries by year - NB
0.4
0.35
0.3
0.25
0.2
0.15
0.1
0.05
0
January
February
March
April
May
June
July
August
September
October
November
December
2012
2013
2014
2015
2016
2017
2018
2019
2020

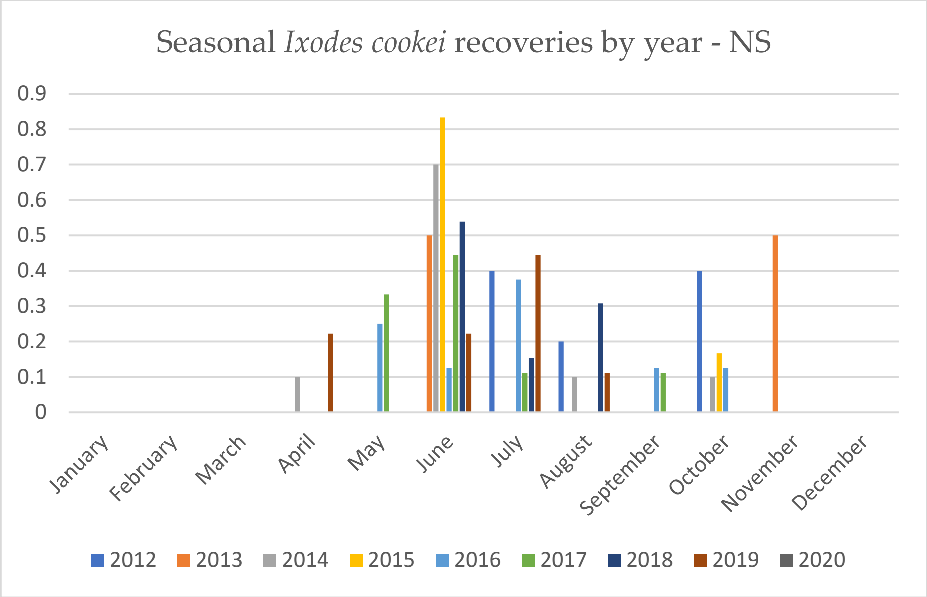
Seasonal Ixodes cookei recoveries by year - NS
0.9
0.8
0.7
0.6
0.5
0.4
0.3
0.2
0.1
0
January
February
March
April
May
June
July
August
September
October
November
December
2012
2013
2014
2015
2016
2017
2018
2019
2020

C.

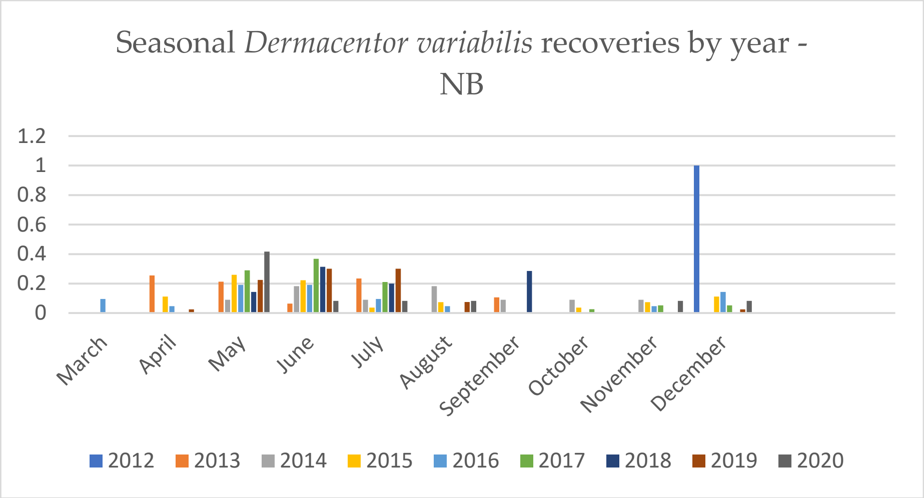
Seasonal Dermacentor variabilis recoveries by year - NB
1.2
1
0.8
0.6
0.4
0.2
0
March
April
May
June
July
August
September
October
November
December
2012
2013
2014
2015
2016
2017
2018
2019
2020

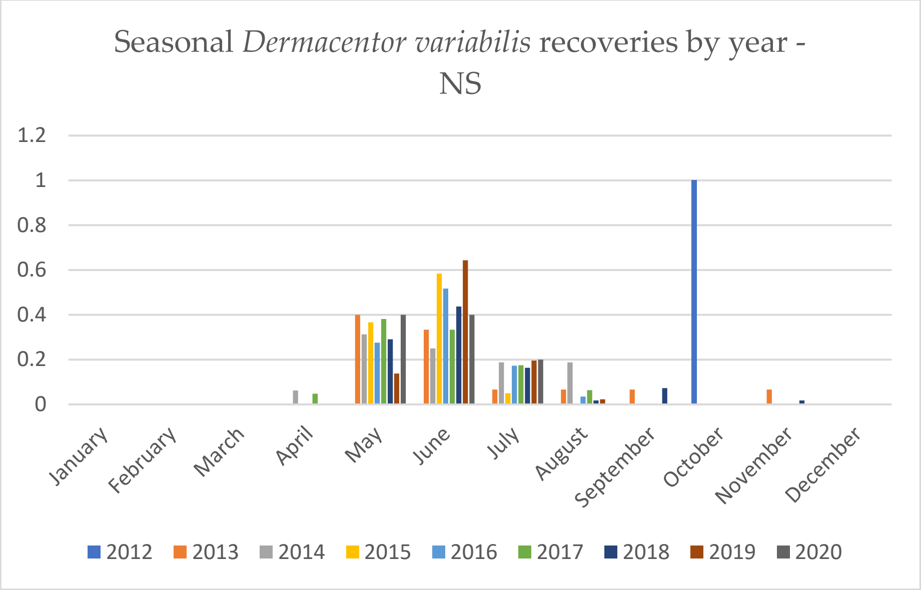

Supplemental Figure S1. Seasonal recoveries of *Ixodes scapularis* (A), *Ixodes cookei* (B) and *Dermacentor variabilis* (C) in New Brunswick (NB), Nova Scotia (NS) and Prince Edward Island (PEI). The proportion of the annual tick submissions, per province, is show by month of collection. Insufficient *Ixodes cookei* and *Dermacentor variabilis* were recovered from Prince Edward Island for comparison.
